# (*E*)-2-Phenyl-*N*-tosyl­non-2-en-4-ynamide

**DOI:** 10.1107/S1600536812048489

**Published:** 2012-12-12

**Authors:** Xiang-Zhen Meng, Xin-Jun Wan

**Affiliations:** a Department of Chemistry and Life Science, Chaohu College, Anhui Province, People’s Republic of China

## Abstract

The mol­ecule of the title compound, C_22_H_23_NO_3_S, adopts an *E* conformation about the C=C bond. The dihedral angle between the benzene rings is 23.79 (5)°. In the crystal, pairs of N—H⋯O hydrogen bonds link the mol­ecules, forming inversion dimers. The terminal butyl group is disordered over two sets of sites in a 0.559 (6):0.441 (6) ratio.

## Related literature

For the synthesis of the titlw compound, see: Cheng *et al.* (2012 ▶). For applications of conjugated enynes, see: Ochiai *et al.* (1999 ▶); Saito *et al.* (2001 ▶).
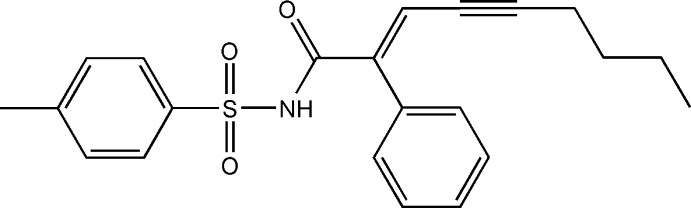


## Experimental

### 

#### Crystal data

C_22_H_23_NO_3_S
*M* *_r_* = 381.47
Triclinic, 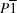

*a* = 9.8186 (10) Å
*b* = 9.8201 (9) Å
*c* = 11.3352 (13) Å
α = 81.470 (8)°
β = 76.308 (9)°
γ = 75.042 (9)°
*V* = 1021.46 (18) Å^3^
*Z* = 2
Mo *K*α radiation
μ = 0.18 mm^−1^
*T* = 293 K
0.42 × 0.38 × 0.32 mm

#### Data collection

Agilent Xcalibur (Atlas, Gemini ultra) diffractometer
Absorption correction: multi-scan (*CrysAlis PRO*; Agilent, 2011 ▶) *T*_min_ = 0.928, *T*_max_ = 0.945
9118 measured reflections
4425 independent reflections
3005 reflections with *I* > 2σ(*I*)
*R*_int_ = 0.028

#### Refinement

*R*[*F* ^2^ > 2σ(*F* ^2^)] = 0.048
*wR*(*F* ^2^) = 0.096
*S* = 1.00
4425 reflections
285 parameters
170 restraints
H-atom parameters constrained
Δρ_max_ = 0.19 e Å^−3^
Δρ_min_ = −0.28 e Å^−3^

### 

Data collection: *CrysAlis PRO* (Agilent, 2011 ▶); cell refinement: *CrysAlis PRO*; data reduction: *CrysAlis PRO*; program(s) used to solve structure: *SHELXTL* (Sheldrick, 2008 ▶); program(s) used to refine structure: *SHELXTL*; molecular graphics: *SHELXTL*; software used to prepare material for publication: *SHELXTL*.

## Supplementary Material

Crystal structure: contains datablock(s) I, global. DOI: 10.1107/S1600536812048489/xu5655sup1.cif

Structure factors: contains datablock(s) I. DOI: 10.1107/S1600536812048489/xu5655Isup2.hkl

Supplementary material file. DOI: 10.1107/S1600536812048489/xu5655Isup3.cml

Additional supplementary materials:  crystallographic information; 3D view; checkCIF report

## Figures and Tables

**Table 1:** Hydrogen-bond geometry (Å, °)

*D*—H⋯*A*	*D*—H	H⋯*A*	*D*⋯*A*	*D*—H⋯*A*
N1—H1⋯O2^i^	0.86	2.32	2.947 (2)	130

Symmetry code: (i) 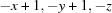.

**Fig. 1.**
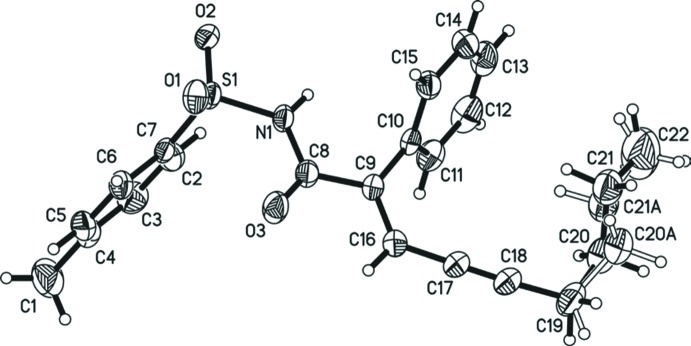
The molecular structure of the compound with displacement ellipsoids drawn at 30% probability level.

**Fig. 2.**
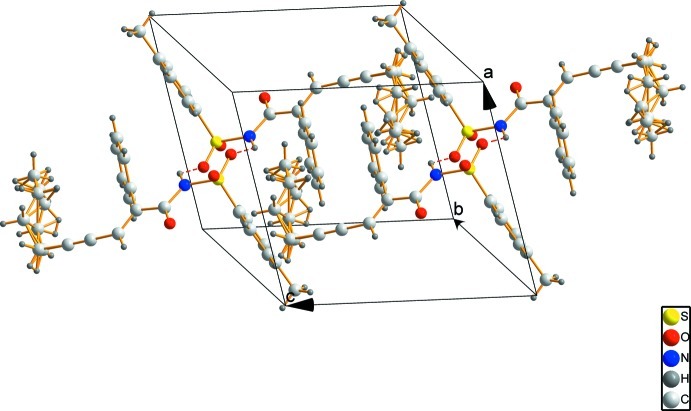
The crystal packing diagram.

## supplementary crystallographic information

### Comment

Conjugated enynes can be used in the synthesis of polymers (Ochiai *et al.*, 1999) and in the selective construction of aromatic frameworks (Saito *et al.*, 2001). Here, we report the crystal structure of the title enyne compound.

The molecular structure of the title compound is shown in Figure 1, the *ORTEP* diagram shows that the structure adopts the E isomer, the double bond and triple bond are within normal ranges. The benzene C2–C7 and C10–C15 rings are tilted relative to each other by 23.79 (5)°. The chain C19—C22 is disorder. A view of the crystal packing for the title compound is illustrated in Fig. 2, the crystal structure is stabilized by N—H···O hydrogen bonds.

### Experimental

The compound was prepared according to the reference (Cheng *et al.*, 2012). 4-Methylbenzenesulfonyl azide (0.45 mmol), CuI (5.7 mg, 0.03 mmol), ethynylbenzene (0.45 mmol), and hept-2-ynal (0.3 mmol) were suspended in THF in a 10 ml Schlenk tube at room temperature at N_2_ atmosphere. Cs_2_CO_3_ (8.64 mg, 0.36 mmol) was then added, and the resulting solution was stirred at this temperature for 24 h. The reaction was quenched by saturated aqueous NH_4_Cl (5 ml) and extracted with CH_2_Cl_2_ (15 ml × 3). The combined organic layers were dried over anhydrous Na_2_SO_4_ and concentrated *in vacuo*. The crude residue was purified by column chromatography on silica gel (n-hexane/EtOAc) to afford the title compound. The title compound was recrystallized from CH_2_Cl_2_ at room temperature to give the desired crystals suitable for single-crystal X-ray diffraction.

### Refinement

H atoms were placed in geometrically idealized positions and constrained to ride on their parent atoms, with aromatic C—H = 0.93–0.97 Å and N—H = 0.86 Å, *U*_iso_(H) = 1.5*U*_eq_(C) for methyl H atoms and 1.2*U*_eq_(C,*N*) for the others. The butyl group is disordered over two positions, site occupancies were refined.

### Crystal data

**Table:**

C_22_H_23_NO_3_S	*Z* = 2
*M**_r_* = 381.47	*F*(000) = 404
Triclinic, *P*1	*D*_x_ = 1.240 Mg m^−^^3^
Hall symbol: -P 1	Mo *K*α radiation, λ = 0.71073 Å
*a* = 9.8186 (10) Å	Cell parameters from 2435 reflections
*b* = 9.8201 (9) Å	θ = 2.9–29.6°
*c* = 11.3352 (13) Å	µ = 0.18 mm^−^^1^
α = 81.470 (8)°	*T* = 293 K
β = 76.308 (9)°	Block, colorless
γ = 75.042 (9)°	0.42 × 0.38 × 0.32 mm
*V* = 1021.46 (18) Å^3^	

### Data collection

**Table:**

Agilent Xcalibur (Atlas, Gemini ultra) diffractometer	4425 independent reflections
Radiation source: fine-focus sealed tube	3005 reflections with *I* > 2σ(*I*)
Graphite monochromator	*R*_int_ = 0.028
Detector resolution: 10.3592 pixels mm^-1^	θ_max_ = 27.1°, θ_min_ = 2.9°
ω scans	*h* = −12→11
Absorption correction: multi-scan (*CrysAlis PRO*; Agilent, 2011)	*k* = −12→12
*T*_min_ = 0.928, *T*_max_ = 0.945	*l* = −14→13
9118 measured reflections	

### Refinement

**Table:**

Refinement on *F*^2^	Secondary atom site location: difference Fourier map
Least-squares matrix: full	Hydrogen site location: inferred from neighbouring sites
*R*[*F*^2^ > 2σ(*F*^2^)] = 0.048	H-atom parameters constrained
*wR*(*F*^2^) = 0.096	*w* = 1/[σ^2^(*F*_o_^2^) + (0.0145*P*)^2^ + 0.450*P*] where *P* = (*F*_o_^2^ + 2*F*_c_^2^)/3
*S* = 1.00	(Δ/σ)_max_ < 0.001
4425 reflections	Δρ_max_ = 0.19 e Å^−^^3^
285 parameters	Δρ_min_ = −0.28 e Å^−^^3^
170 restraints	Extinction correction: *SHELXTL* (Sheldrick, 2008), Fc^*^=kFc[1+0.001xFc^2^λ^3^/sin(2θ)]^-1/4^
Primary atom site location: structure-invariant direct methods	Extinction coefficient: 0.0511 (16)

### Special details

**Table:**

Geometry. All e.s.d.'s (except the e.s.d. in the dihedral angle between two l.s. planes) are estimated using the full covariance matrix. The cell e.s.d.'s are taken into account individually in the estimation of e.s.d.'s in distances, angles and torsion angles; correlations between e.s.d.'s in cell parameters are only used when they are defined by crystal symmetry. An approximate (isotropic) treatment of cell e.s.d.'s is used for estimating e.s.d.'s involving l.s. planes.
Refinement. Refinement of *F*^2^ against ALL reflections. The weighted *R*-factor *wR* and goodness of fit *S* are based on *F*^2^, conventional *R*-factors *R* are based on *F*, with *F* set to zero for negative *F*^2^. The threshold expression of *F*^2^ > σ(*F*^2^) is used only for calculating *R*-factors(gt) *etc*. and is not relevant to the choice of reflections for refinement. *R*-factors based on *F*^2^ are statistically about twice as large as those based on *F*, and *R*- factors based on ALL data will be even larger.

### Fractional atomic coordinates and isotropic or equivalent isotropic displacement parameters (Å^2^)

**Table:**

	*x*	*y*	*z*	*U*_iso_*/*U*_eq_	Occ. (<1)
S1	0.49748 (6)	0.27503 (5)	0.07181 (5)	0.04966 (17)	
O1	0.56428 (17)	0.13893 (14)	0.11958 (14)	0.0656 (4)	
O2	0.58196 (15)	0.35117 (14)	−0.02248 (13)	0.0575 (4)	
O3	0.3159 (2)	0.23691 (16)	0.31525 (14)	0.0779 (5)	
N1	0.43740 (19)	0.38162 (16)	0.18182 (15)	0.0518 (5)	
H1	0.4617	0.4614	0.1702	0.062*	
C1	−0.0183 (3)	0.2430 (4)	−0.1135 (4)	0.1257 (13)	
H1A	−0.1011	0.2483	−0.0478	0.189*	
H1B	0.0011	0.1555	−0.1495	0.189*	
H1C	−0.0368	0.3210	−0.1741	0.189*	
C2	0.2639 (3)	0.3857 (2)	−0.0279 (2)	0.0631 (6)	
H2	0.2878	0.4724	−0.0331	0.076*	
C3	0.1480 (3)	0.3764 (3)	−0.0711 (2)	0.0757 (7)	
H3	0.0932	0.4579	−0.1053	0.091*	
C4	0.1106 (3)	0.2494 (3)	−0.0651 (2)	0.0753 (8)	
C5	0.1929 (3)	0.1314 (3)	−0.0146 (3)	0.0774 (8)	
H5	0.1695	0.0446	−0.0105	0.093*	
C6	0.3097 (3)	0.1372 (2)	0.0305 (2)	0.0608 (6)	
H6	0.3638	0.0557	0.0652	0.073*	
C7	0.3449 (2)	0.26491 (19)	0.02339 (17)	0.0464 (5)	
C8	0.3496 (2)	0.3491 (2)	0.29221 (19)	0.0529 (5)	
C9	0.3001 (2)	0.4641 (2)	0.37598 (18)	0.0483 (5)	
C10	0.3535 (2)	0.5957 (2)	0.34604 (17)	0.0461 (5)	
C11	0.2602 (3)	0.7245 (2)	0.3255 (2)	0.0645 (6)	
H11	0.1639	0.7281	0.3285	0.077*	
C12	0.3082 (4)	0.8474 (3)	0.3008 (2)	0.0830 (9)	
H12	0.2442	0.9333	0.2871	0.100*	
C13	0.4484 (4)	0.8444 (3)	0.2964 (2)	0.0825 (9)	
H13	0.4803	0.9278	0.2790	0.099*	
C14	0.5422 (3)	0.7185 (3)	0.3175 (2)	0.0743 (7)	
H14	0.6379	0.7163	0.3158	0.089*	
C15	0.4949 (3)	0.5942 (2)	0.3415 (2)	0.0576 (6)	
H15	0.5597	0.5086	0.3547	0.069*	
C16	0.2024 (3)	0.4427 (2)	0.4767 (2)	0.0615 (6)	
H16	0.1715	0.3588	0.4888	0.074*	
C17	0.1426 (3)	0.5407 (3)	0.5667 (2)	0.0658 (7)	
C18	0.0934 (3)	0.6244 (3)	0.6390 (2)	0.0734 (7)	
C19	0.0400 (14)	0.7233 (10)	0.7344 (10)	0.096 (4)	0.559 (6)
H19A	0.0823	0.6839	0.8048	0.115*	0.559 (6)
H19B	−0.0639	0.7386	0.7600	0.115*	0.559 (6)
C20	0.0816 (6)	0.8656 (7)	0.6826 (7)	0.099 (2)	0.559 (6)
H20A	0.0368	0.9339	0.7422	0.119*	0.559 (6)
H20B	0.0427	0.9008	0.6100	0.119*	0.559 (6)
C21	0.2508 (6)	0.8559 (7)	0.6491 (7)	0.0798 (19)	0.559 (6)
H21A	0.2915	0.8123	0.7195	0.096*	0.559 (6)
H21B	0.2945	0.7949	0.5837	0.096*	0.559 (6)
C22	0.2860 (10)	0.9846 (8)	0.6137 (9)	0.121 (3)	0.559 (6)
H22A	0.3887	0.9717	0.5986	0.181*	0.559 (6)
H22B	0.2416	1.0465	0.6772	0.181*	0.559 (6)
H22C	0.2521	1.0257	0.5406	0.181*	0.559 (6)
C19A	0.0304 (13)	0.7466 (14)	0.7125 (13)	0.084 (3)	0.441 (6)
H19C	−0.0326	0.7186	0.7869	0.101*	0.441 (6)
H19D	−0.0269	0.8220	0.6670	0.101*	0.441 (6)
C20A	0.1514 (10)	0.8022 (9)	0.7455 (6)	0.096 (2)	0.441 (6)
H20C	0.2342	0.7266	0.7574	0.115*	0.441 (6)
H20D	0.1162	0.8556	0.8164	0.115*	0.441 (6)
C21A	0.1841 (16)	0.8992 (12)	0.6240 (9)	0.124 (3)	0.441 (6)
H21C	0.2709	0.8427	0.5777	0.149*	0.441 (6)
H21D	0.1080	0.8986	0.5831	0.149*	0.441 (6)
C22A	0.2024 (16)	1.0297 (11)	0.5965 (14)	0.160 (5)	0.441 (6)
H22D	0.2938	1.0283	0.5419	0.239*	0.441 (6)
H22E	0.2000	1.0693	0.6697	0.239*	0.441 (6)
H22F	0.1266	1.0864	0.5580	0.239*	0.441 (6)

### Atomic displacement parameters (Å^2^)

**Table:**

	*U*^11^	*U*^22^	*U*^33^	*U*^12^	*U*^13^	*U*^23^
S1	0.0597 (3)	0.0411 (3)	0.0472 (3)	−0.0092 (2)	−0.0053 (3)	−0.0148 (2)
O1	0.0807 (11)	0.0439 (8)	0.0695 (11)	0.0011 (7)	−0.0232 (9)	−0.0118 (7)
O2	0.0599 (9)	0.0561 (8)	0.0546 (9)	−0.0175 (7)	0.0046 (7)	−0.0182 (7)
O3	0.1151 (15)	0.0594 (9)	0.0603 (10)	−0.0392 (10)	0.0058 (10)	−0.0144 (8)
N1	0.0683 (12)	0.0441 (9)	0.0449 (10)	−0.0186 (8)	−0.0025 (9)	−0.0154 (8)
C1	0.081 (2)	0.166 (3)	0.153 (3)	−0.021 (2)	−0.040 (2)	−0.074 (3)
C2	0.0676 (16)	0.0556 (13)	0.0668 (16)	−0.0143 (12)	−0.0147 (13)	−0.0064 (11)
C3	0.0676 (17)	0.0838 (18)	0.0725 (18)	−0.0055 (14)	−0.0183 (14)	−0.0122 (14)
C4	0.0601 (16)	0.099 (2)	0.0725 (17)	−0.0169 (15)	−0.0041 (14)	−0.0429 (16)
C5	0.0717 (18)	0.0737 (17)	0.094 (2)	−0.0262 (15)	−0.0009 (16)	−0.0413 (15)
C6	0.0693 (16)	0.0480 (12)	0.0651 (15)	−0.0148 (11)	−0.0040 (12)	−0.0193 (11)
C7	0.0553 (13)	0.0445 (11)	0.0385 (11)	−0.0129 (9)	0.0000 (9)	−0.0141 (9)
C8	0.0640 (14)	0.0505 (12)	0.0462 (12)	−0.0160 (11)	−0.0087 (11)	−0.0104 (10)
C9	0.0536 (13)	0.0529 (11)	0.0409 (12)	−0.0134 (10)	−0.0089 (10)	−0.0116 (9)
C10	0.0568 (13)	0.0477 (11)	0.0338 (10)	−0.0093 (10)	−0.0079 (9)	−0.0112 (8)
C11	0.0732 (16)	0.0630 (14)	0.0511 (14)	−0.0033 (12)	−0.0158 (12)	−0.0035 (11)
C12	0.117 (3)	0.0498 (14)	0.0658 (17)	−0.0027 (15)	−0.0091 (17)	0.0021 (12)
C13	0.131 (3)	0.0585 (16)	0.0590 (16)	−0.0407 (18)	0.0002 (17)	−0.0082 (12)
C14	0.0855 (19)	0.0792 (18)	0.0674 (17)	−0.0391 (15)	−0.0066 (14)	−0.0155 (14)
C15	0.0634 (15)	0.0532 (12)	0.0585 (14)	−0.0144 (11)	−0.0116 (12)	−0.0122 (10)
C16	0.0682 (15)	0.0689 (14)	0.0531 (14)	−0.0278 (12)	−0.0043 (12)	−0.0164 (11)
C17	0.0645 (15)	0.0796 (16)	0.0535 (14)	−0.0271 (13)	0.0059 (12)	−0.0179 (13)
C18	0.0740 (17)	0.0854 (17)	0.0586 (15)	−0.0279 (14)	0.0098 (13)	−0.0216 (14)
C19	0.118 (6)	0.085 (5)	0.071 (5)	−0.028 (4)	0.022 (4)	−0.029 (4)
C20	0.101 (4)	0.097 (4)	0.096 (5)	−0.024 (4)	0.005 (4)	−0.041 (4)
C21	0.089 (4)	0.089 (4)	0.066 (3)	−0.018 (3)	−0.024 (3)	−0.016 (3)
C22	0.143 (7)	0.104 (6)	0.130 (6)	−0.056 (5)	−0.011 (6)	−0.037 (5)
C19A	0.095 (6)	0.092 (6)	0.058 (5)	−0.026 (5)	0.018 (4)	−0.030 (5)
C20A	0.117 (6)	0.110 (5)	0.063 (4)	−0.030 (4)	−0.006 (4)	−0.031 (4)
C21A	0.139 (7)	0.145 (7)	0.099 (6)	−0.068 (6)	0.015 (6)	−0.042 (5)
C22A	0.197 (11)	0.108 (7)	0.121 (8)	0.018 (8)	−0.002 (9)	0.009 (6)

### Geometric parameters (Å, º)

**Table:**

S1—O1	1.4179 (15)	C14—C15	1.384 (3)
S1—O2	1.4329 (14)	C14—H14	0.9300
S1—N1	1.6488 (15)	C15—H15	0.9300
S1—C7	1.743 (2)	C16—C17	1.424 (3)
O3—C8	1.205 (2)	C16—H16	0.9300
N1—C8	1.387 (3)	C17—C18	1.178 (3)
N1—H1	0.8600	C18—C19	1.472 (6)
C1—C4	1.512 (4)	C18—C19A	1.479 (7)
C1—H1A	0.9600	C19—C20	1.550 (8)
C1—H1B	0.9600	C19—H19A	0.9700
C1—H1C	0.9600	C19—H19B	0.9700
C2—C3	1.368 (3)	C20—C21	1.595 (6)
C2—C7	1.380 (3)	C20—H20A	0.9700
C2—H2	0.9300	C20—H20B	0.9700
C3—C4	1.377 (3)	C21—C22	1.374 (6)
C3—H3	0.9300	C21—H21A	0.9700
C4—C5	1.365 (4)	C21—H21B	0.9700
C5—C6	1.378 (3)	C22—H22A	0.9600
C5—H5	0.9300	C22—H22B	0.9600
C6—C7	1.371 (3)	C22—H22C	0.9600
C6—H6	0.9300	C19A—C20A	1.567 (9)
C8—C9	1.493 (3)	C19A—H19C	0.9700
C9—C16	1.335 (3)	C19A—H19D	0.9700
C9—C10	1.482 (3)	C20A—C21A	1.570 (8)
C10—C15	1.373 (3)	C20A—H20C	0.9700
C10—C11	1.382 (3)	C20A—H20D	0.9700
C11—C12	1.373 (3)	C21A—C22A	1.321 (8)
C11—H11	0.9300	C21A—H21C	0.9700
C12—C13	1.358 (4)	C21A—H21D	0.9700
C12—H12	0.9300	C22A—H22D	0.9600
C13—C14	1.365 (4)	C22A—H22E	0.9600
C13—H13	0.9300	C22A—H22F	0.9600
			
O1—S1—O2	118.88 (10)	C15—C14—H14	120.0
O1—S1—N1	109.36 (9)	C10—C15—C14	120.8 (2)
O2—S1—N1	103.78 (8)	C10—C15—H15	119.6
O1—S1—C7	109.46 (9)	C14—C15—H15	119.6
O2—S1—C7	109.04 (9)	C9—C16—C17	123.9 (2)
N1—S1—C7	105.40 (9)	C9—C16—H16	118.1
C8—N1—S1	123.31 (13)	C17—C16—H16	118.1
C8—N1—H1	118.3	C18—C17—C16	178.4 (3)
S1—N1—H1	118.3	C17—C18—C19	175.8 (7)
C4—C1—H1A	109.5	C17—C18—C19A	170.7 (8)
C4—C1—H1B	109.5	C19—C18—C19A	12.7 (12)
H1A—C1—H1B	109.5	C18—C19—C20	108.9 (6)
C4—C1—H1C	109.5	C18—C19—H19A	109.9
H1A—C1—H1C	109.5	C20—C19—H19A	109.9
H1B—C1—H1C	109.5	C18—C19—H19B	109.9
C3—C2—C7	119.2 (2)	C20—C19—H19B	109.9
C3—C2—H2	120.4	H19A—C19—H19B	108.3
C7—C2—H2	120.4	C19—C20—C21	114.3 (9)
C2—C3—C4	121.6 (3)	C19—C20—H20A	108.7
C2—C3—H3	119.2	C21—C20—H20A	108.7
C4—C3—H3	119.2	C19—C20—H20B	108.7
C5—C4—C3	118.0 (2)	C21—C20—H20B	108.7
C5—C4—C1	121.8 (3)	H20A—C20—H20B	107.6
C3—C4—C1	120.2 (3)	C22—C21—C20	113.8 (6)
C4—C5—C6	121.8 (2)	C22—C21—H21A	108.8
C4—C5—H5	119.1	C20—C21—H21A	108.8
C6—C5—H5	119.1	C22—C21—H21B	108.8
C7—C6—C5	119.1 (2)	C20—C21—H21B	108.8
C7—C6—H6	120.5	H21A—C21—H21B	107.7
C5—C6—H6	120.5	C18—C19A—C20A	110.9 (8)
C6—C7—C2	120.2 (2)	C18—C19A—H19C	109.5
C6—C7—S1	120.43 (17)	C20A—C19A—H19C	109.5
C2—C7—S1	119.29 (16)	C18—C19A—H19D	109.5
O3—C8—N1	121.30 (18)	C20A—C19A—H19D	109.5
O3—C8—C9	124.1 (2)	H19C—C19A—H19D	108.0
N1—C8—C9	114.59 (17)	C19A—C20A—C21A	97.2 (11)
C16—C9—C10	122.48 (18)	C19A—C20A—H20C	112.3
C16—C9—C8	116.03 (19)	C21A—C20A—H20C	112.3
C10—C9—C8	121.45 (17)	C19A—C20A—H20D	112.3
C15—C10—C11	118.2 (2)	C21A—C20A—H20D	112.3
C15—C10—C9	121.44 (19)	H20C—C20A—H20D	109.9
C11—C10—C9	120.4 (2)	C22A—C21A—C20A	135.1 (11)
C12—C11—C10	120.7 (3)	C22A—C21A—H21C	103.4
C12—C11—H11	119.6	C20A—C21A—H21C	103.4
C10—C11—H11	119.6	C22A—C21A—H21D	103.4
C13—C12—C11	120.5 (3)	C20A—C21A—H21D	103.4
C13—C12—H12	119.8	H21C—C21A—H21D	105.2
C11—C12—H12	119.8	C21A—C22A—H22D	109.5
C12—C13—C14	119.8 (2)	C21A—C22A—H22E	109.5
C12—C13—H13	120.1	H22D—C22A—H22E	109.5
C14—C13—H13	120.1	C21A—C22A—H22F	109.5
C13—C14—C15	120.0 (3)	H22D—C22A—H22F	109.5
C13—C14—H14	120.0	H22E—C22A—H22F	109.5

### Hydrogen-bond geometry (Å, º)

**Table:**

*D*—H···*A*	*D*—H	H···*A*	*D*···*A*	*D*—H···*A*
N1—H1···O2^i^	0.86	2.32	2.947 (2)	130

Symmetry code: (i) −*x*+1, −*y*+1, −*z*.
